# Care priorities for stroke patients developing cognitive difficulties: a Delphi survey of UK professional views

**DOI:** 10.1186/s12913-020-05558-y

**Published:** 2020-08-05

**Authors:** Eugene Y. H. Tang, Louise Robinson, Catherine Exley, Darren Flynn, Blossom C. M. Stephan, Christopher Price

**Affiliations:** 1 Population Health Sciences Institute, Newcastle University, Campus for Ageing and Vitality, Level 2, Newcastle Biomedical Research Building, Newcastle upon Tyne, NE4 5PL UK; 2 Centre for Rehabilitation, Exercise and Sports Science, School of Health & Life Sciences, Teesside University, Middlesbrough Tees Valley, TS1 3BX UK; 3 Division of Psychiatry and Applied Psychology, Institute of Mental Health, School of Medicine, University of Nottingham, Innovation Park, Nottingham, NG7 2TU UK

**Keywords:** Stroke, Cognition, Risk assessment, Dementia, Delphi

## Abstract

**Background:**

Post stroke cognitive difficulties are common but generally prioritised below other impairments. In the UK, clinical guidelines recommend a holistic review at six-months post-stroke including an assessment of cognitive function. In order to assist clinicians to provide better care for patients with post-stroke cognitive deficits and assist with service planning, our aim was to establish professional consensus on key actions at the six-month review.

**Methods:**

An electronic Delphi survey was developed with ten potential actions for clinicians to prioritise across five different clinical scenarios describing patients with cognitive difficulties. Scenarios varied in terms of age of the stroke-survivor, stroke severity and use of dementia risk assessment. A panel of professional volunteers was obtained through the British Association of Stroke Physicians and the UK National Stroke Nursing Forum.

**Results:**

Forty-five stroke clinicians completed round one, with 21 participants completing round two. Priorities consistently supported by professionals included access to psychological services, screening for a mood disorder and ensuring multi-professional input. Direct access to specialist memory services was not generally supported unless a dementia risk assessment tool indicated that the individual was at high risk of dementia.

**Conclusions:**

Assessment of post-stroke cognitive deficits needs to be routinely considered during the six-month review. A formal risk assessment tool could be a way to streamline direct access to memory clinic services to ensure that individuals at-risk of dementia receive ongoing care.

## Background

Stroke is a leading global cause of mortality, disability and high economic burden due to the costs of treatment and subsequent care [1]. In the United Kingdom (UK), the national healthcare strategy has identified stroke as a clinical priority and aims to improve rehabilitation for stroke-survivors upon discharge [2]. Although the focus here is often on physical recovery, in the first-year post-stroke 4 in 10 patients display some degree of cognitive impairment without a diagnosis of dementia [3]. This can be linked to demographic and illness factors. Around 6 months post-stroke, females with a history of cerebrovascular disease and those who had either a lacunar or posterior circulation infarct are more likely to have developed a new cognitive impairment [4]. The identification of dementia is also more challenging due to additional persistent deficits post-stroke, both with global cognition and individual domains e.g. attention and processing speed, memory, language and frontal executive function can be affected [5]. A history of stroke is also a strong independent risk factor for dementia [6]. It accelerates the onset of dementia by 10 years [7] and 10% of individuals develop dementia soon after their first stroke [8].

National Clinical Guidelines recommend clinical neuropsychology or clinical psychology provision for severe or persistent disturbance in cognitive function after stroke, with routine follow-up [9]. As part of long-term post-stroke care, the National Stroke strategy had previously recommended that all stroke survivors should have a six-month review [10] although the clinician conducting these reviews can vary [11]. This was further emphasised in care guidelines produced by the National Institute for Health and Care Excellence [12]. The latest national audit of clinical services via the Sentinel Stroke National Audit Programme, reports that these reviews are conducted by a stroke coordinator (32.8%), therapist (10%), secondary care clinician (10.5%), district/community nurse (10%), a General Practitioner (0.1%), voluntary services employee (9.9%) or others (26.7%) [13]. At the six-month review, clinicians are encouraged to enquire about any cognition problems. However, a national audit of post-acute services found that there are still a number of areas where 6-month reviews are not being performed [14]. Further, a survey was conducted by a UK charity, the Stroke Association of 1424 stroke survivors from across England who detailed their own personal experiences of stroke care which was carried out between January to March 2016. They found that 77% of stroke survivors have problems with their memory with nearly 50% rating the support they received for memory problems and fatigue as poor [15].

In order to assist clinicians and service planners in providing targeted care for stroke-survivors with subsequent new cognitive issues, we sought professional views about areas of high and low priority during the six-month review. We chose this time point as it would be more likely to reflect long-term and stable post-stroke sequelae as opposed to a shorter time where deficits could be due to the immediate impact of a stroke illness. As current assessment guidance remains generic, we attempted to ascertain whether care priorities should reflect differing patient characteristics.

## Methods

### Delphi participants

Two professional societies, the British Association of Stroke Physicians (BASP) and the National Stroke Nursing Forum (NSNF) agreed to disseminate the Delphi survey to their members. Administrators of both organisations sent out the initial invitation email to their members on behalf of the research team. Stroke physicians (defined as any physician involved in stroke care) and stroke nurses currently employed with NHS stroke clinical services were eligible to participate. Recipients were also asked to forward the survey details to any relevant Allied Health Professional groups they were connected to. Initial contact was through BASP or NSNF with email addresses provided to the research team by participants after round one. Subsequent contact with participants was then from the research team directly for round 2. We aimed to achieve broad geographical and professional coverage.

### Questionnaire

The options were based on previous findings from our qualitative study [16] and discussion amongst the research team. Demographic data was collected including age, clinical role and years of experience in that role. The survey was case-based and asked participants to rate the extent that they approved or disapproved of 10 potential actions that could be carried out in common clinical scenarios occurring at the six-month clinic review (see Table 1). Although the options remained the same in each case, the clinical scenario content would vary. The scenarios were informed by previous work and generated through discussion amongst the research team which consists of General Practitioners (ET and LR), a senior stroke clinician (CP) and two researchers with experience of working in stroke and mental health using qualitative and preference elicitation approaches (CE and DF). The first three scenarios looked at issues related to the stroke-survivor themselves e.g. in general, for the young stroke-survivor and severity of the stroke. The final two scenarios incorporated the concept of risk assessment for dementia [17, 18] to assess whether use of such a tool would change clinical priorities. The scenarios described use of a tool which could identify an individual being at high or low risk of a future dementia. Participants were asked to use their judgement to consider the availability, practicality and cost effectiveness of each option. The survey was distributed via the Online Surveys platform (www.onlinesurveys.ac.uk).

**Table 1:** Clinical Scenarios and Available Actions to be Ranked

Clinical Scenarios Presented to Each Delphi Participant in Round 1	Clinical Actions to be Ranked For Each Scenario
CASE 1: Please prioritise the following actions for all stroke-survivors presenting with new post-stroke cognitive deficits reported at their six month review	Access to psychological services Additional communication with the GP Cognitive screen e.g. MoCA during six-month stroke clinic review Direct access to memory clinic services Ensuring allied health professional community follow-up e.g. occupational therapist for additional follow-up review in the community Ensuring compliance to secondary prevention is in place Follow-up in stroke-services GP to perform cognitive screen following discharge from specialist services Screening for a mood disorder Signposting individuals to other sources of information e.g. Stroke Association
CASE 2: Please prioritise the following actions for young stroke-survivors (under the age of 60) who are currently working presenting with new post-stroke cognitive deficits at their six-month review	
CASE 3: Please prioritise the following actions for stroke-survivors presenting with new post-stroke cognitive deficits after a severe stroke resulting in dependence on others at their six-month review.	
CASE 4: Please consider the following scenario. A 72 year stroke-survivor who presents with new subjective memory complaints. On further assessment using a risk prediction tool, she was found to be at high risk of developing cognitive failure/dementia in the next 2 years. Please prioritise the actions below based on this scenario.	
CASE 5: Please consider the following scenario. A 67 year stroke-survivor who presents with new subjective memory complaints. On a screening assessment using a risk prediction tool, he was found to be at low risk of developing cognitive failure/dementia in the next 2 years. Please prioritise the actions below based on this scenario.	

### Data collection

In the first Delphi round participants were presented with five clinical scenarios (see Table 1) and asked to assign ratings using a 7-point Likert scale (from very strongly disapprove to very strongly approve) to each of the ten options. Participants were also given an opportunity to provide free text comments. The overall rating assigned to each statement in round one was dependent on the median and interquartile range (IQR). If the statement had the same median and IQR they were ranked according to the total percentage of individuals who approved, quite strongly approved or very strongly approved the option. Finally, if any statements were still equal ranked after applying these first two criteria, the percentage of those who very strongly approved was used as the deciding factor for ranking. After round one, panellists were informed of summary statistics including aggregate median and the options were presented in rank order. Each participant was also reminded of the individual ratings they had given to all the options in the previous round and their free text comments.

Following discussions amongst the research team, given the consistent levels of approval ratings across the clinical scenarios for each statement in round one, a ranking exercise approach was then used to gain the final overall prioritisation for each statement. Participants were asked to rank the 10 options from 1 (most important) to 10 (least important) for each scenario. The overall ranking in round two was based on a points system for each rank. For example, 10 points were allocated for the statement ranked first (most important), 9 points for the statement for the second ranked statement and so on. The points for each statement in each scenario were then totalled and the ranking of each statement in each scenario was determined by the overall score from all respondents.

For each round, we allowed 2 weeks for participants to respond before a reminder was sent out with an additional week given to complete the survey before the survey was closed.

### Data analysis

In line with other studies, consensus was defined as achieved if there was ≥75% agreement of all replies to a statement that fell within three categories (approve, quite or very strongly approve or disapprove) on the Likert scale [19–21]. The data was transferred and analysed using *Excel and STATA 15/16.*

## Results

### Round one

The demographics of the panellists that participated in round one is described in Table 2. In total there were 45 individuals, the majority were female (68.9%). There was representation from stroke physicians (44.4%; including neurologists), stroke nurses (46.7%; including specialist nurses (*n* = 13), stroke nurse (*n* = 6), stroke research nurse (*n* = 1) and stroke nurse practitioner (*n* = 1)) and allied health professionals (8.9%; including speech and language therapist, occupational therapists and physiotherapist). There was representation from most areas of the UK, except for East of England in round 1. The majority of participants in round one also reported that they performed six-month reviews (64.4%). Consensus was agreed to approve the majority of the statements in each clinical scenario (see Table 3). The only statement that consistently did not meet the consensus approval benchmark for the majority of the clinical scenarios was “GP to perform cognitive screen following discharge from specialist services”. Responses in detail can be found in the online supplementary Table 1.

**Table 2:** Demographics of Participants

	Round 1 (***n*** = 45) (n (%))	Round 2 (***n*** = 21) (n (%))
**Age**		
21–30	3 (6.7)	0 (0)
31–40	10 (22.2)	4 (19.1)
41–50	16 (35.6)	9 (42.9)
51–60	10 (22.2)	3 (14.3)
61–70	6 (13.3)	5 (23.8)
**Gender**		
Female	31 (68.9)	15 (71.4)
Male	13 (28.9)	6 (28.6)
Prefer not to say	1 (2.2)	0 (0)
**Geographical Area**		
East Midlands	2 (4.4)	2 (9.5)
Isle of Man	1 (2.2)	1 (4.8)
Isle of Wight	1 (2.2)	0 (0)
London	2 (4.4)	1 (4.8)
North East England	6 (13.3)	3 (14.3)
North West England	2 (4.4)	0 (0)
Oxford/Thames Valley	1 (2.2)	0 (0)
Scotland	7 (15.6)	3 (14.3)
South East England	6 (13.3)	1 (4.8)
South West England	6 (13.3)	3 (14.3)
Wales	3 (6.7)	2 (9.5)
West Midlands	5 (11.1)	3 (14.3)
Yorkshire and the Humber	3 (6.7)	2 (9.5)
**Years of Experience**		
0–5 years	13 (28.9)	1 (4.8)
6–10 years	9 (20.0)	6 (28.6)
11 or more years	23 (51.1)	14 (66.7)
**Role**		
Physician	20 (44.4)	12 (57.1)
Allied Health Professional	4 (8.9)	2 (9.5)
Nurse	21 (46.7)	7 (33.3)
**Currently Performs Six-Month Review**		
No	16 (35.6)	10 (47.6)
Yes	29 (64.4)	11 (52.4)

**Table 3:** Summary Statistics Approval Ratings of Round 1

	Case 1		Case 2		Case 3		Case 4		Case 5	
Statement	Median (IQR)	% Approval and Above	Median (IQR)	% Approval and Above	Median (IQR)	% Approval and Above	Median (IQR)	% Approval and Above	Median (IQR)	% Approval and Above
Access to psychological services	7 (2)	91.1	7 (0)	97.8	5 (1)	82.2	6 (2)	95.6	5 (2)	64.4
Additional communication with the GP	6 (1)	84.4	6 (2)	91.1	5 (2)	84.4	6 (2)	93.3	5 (1)	80.0
Cognitive screen e.g. MoCA during six-month stroke clinic review	5 (2)	75.6	6 (2)	80.0	5 (2)	62.2	6 (1)	91.1	5 (2)	80.0
Direct access to memory clinic services	5 (1)	82.2	5 (2)	80.0	5 (2)	66.7	7 (1)	91.1	5 (2)	55.6
Ensuring allied health professional community follow-up e.g. occupational therapist for additional follow-up review in the community	6 (2)	86.7	7 (1)	91.1	6 (2)	88.9	6 (2)	84.4	5 (2)å	60.0
Ensuring compliance to secondary prevention is in place	6 (2)	91.1	7 (1)	97.8	6 (2)	93.3	7 (1)	97.8	6 (2)	93.3
Follow-up in stroke-services	6 (2)	77.8	6 (2)	88.9	6 (3)	68.9	5 (2)	71.1	5 (2)	66.7
GP to perform cognitive screen following discharge from specialist services	4 (1)	46.7	5 (1)	55.6	4 (1)	46.7	5 (1)	75.6	5 (1)	68.9
Screening for a mood disorder	6 (2)	95.6	7 (1)	97.8	6 (2)	95.6	6 (1)	100.0	6 (2)	93.3
Signposting individuals to other sources of information e.g. Stroke Association	7 (1)	97.8	7 (1)	97.8	7 (1)	95.6	6 (1)	95.6	6 (2)	95.6

**Key:** very strongly disapprove (1), quite strongly disapprove (2), disapprove (3), neutral (4), approve (5), quite strongly approve (6), very strongly approve (7)

### Round two

The demographics of round two participants are described in Table 2. Out of the 45 participants, 43 participants provided an email address to be contacted again for round two. Out of the 43, there were 21 eligible responses including stroke nurses (stroke nurse practitioner (*n* = 1), stroke nurse (*n* = 1), stroke specialist nurse (*n* = 5)). There was good geographical coverage with a mixture of participants from all three clinical groups represented, although there were more physician responses (57.1%). Approximately half of the participants conducted six-month stroke reviews (52.4%).

Table 4 describes the ranking of the actions in each case in both rounds 1 and 2. When it came to prioritising actions for stroke-survivors (including young stroke-survivors and also those with severe stroke resulting in dependence) presenting with cognitive difficulties, panellists felt that screening for a mood disorder was consistently a high priority across all three scenarios. Review by allied health professional in the community was also felt to be important to stroke-survivors presenting with cognitive deficits, again irrespective of the age or severity of problems. Finally, access to psychological services was important particularly for the young stroke survivor with cognitive deficits. There was limited agreement on the role of primary care, as the proposal for General Practitioner’s (GPs) to perform cognitive screening was ranked consistently low across all scenarios presented to the panellists. Direct access to memory clinic services was ranked consistently low across the first three scenarios. Although additional follow-up in stroke services was not generally approved across the scenarios, it was approved as a top three action for those with severe stroke and cognitive difficulties.

**Table 4:** Ranking of Each Statement by Case in Rounds 1 and 2

	Case 1		Case 2		Case 3		Case 4		Case 5	
Statement	Round 1	Round 2	Round 1	Round 2	Round 1	Round 2	Round 1	Round 2	Round 1	Round 2
Access to psychological services	2	1	1	1	6	4	6	5	8	7
Additional communication with the GP	3	4	6	8	7	7	7	6	4	6
Cognitive screen e.g. MoCA during six-month stroke clinic review	9	6	8	4	9	8	5	2	6	4
Direct access to memory clinic services	8	8	10	9	8	9	2	1	10	10
Ensuring allied health professional community follow-up e.g. occupational therapist for additional follow-up review in the community	6	3	5	2	4	1	8	7	9	8
Ensuring compliance to secondary prevention is in place	5	9	4	6	3	6	1	3	2	3
Follow-up in stroke-services	7	7	7	5	5	3	10	9	7	5
GP to perform cognitive screen following discharge from specialist services	10	10	9	10	10	10	9	10	5	9
Screening for a mood disorder	4	2	3	3	2	2	3	4	3	1
Signposting individuals to other sources of information e.g. Stroke Association	1	5	2	7	1	5	4	8	1	2

In the scenarios where a risk assessment for dementia was applied, irrespective of whether the individual was at high or low risk, respondents felt that screening for a mood disorder and compliance with secondary prevention were important. Unlike the previous scenarios, if an individual was found to be at high risk then direct access to memory clinics and a cognitive screen at the six-month review were felt to be important actions. Responses in detail can be found in the online supplementary Table 2.

## Discussion

It is recommended that all stroke survivors receive a six-month review including a cognitive assessment, but there is no standardisation of content for general or specific patient groups. This exploratory electronic Delphi exercise by practicing National Health Service stroke clinicians and nurses describes priorities for actions at the six-month review. Irrespective of age and severity of stroke, there was agreement that actions should include screening for a mood disorder, ensuring allied health professional follow-up and access to psychology. It remains unclear which service should provide ongoing care as follow-up in stroke services was inconsistently approved and GP input in the form of cognitive assessment was consistently disapproved. However, direct access to memory clinics was approved if a risk assessment tool was used to identify individuals that were at high risk of developing dementia. This might reflect clinicians’ views that earlier intervention delays or even reduces future cognitive decline, and that patients and carers have specialist information and support needs that cannot be met by standard stroke services.

### Strengths and limitations

We captured the opinions of professionals involved in stroke care across the UK and professional groups but recognise several limitations. Although the response options available were generated and informed through discussion within an experienced research team and based on evidence from our previous work, only limited options were available to the participants to rank in each round. The fact that we were able to achieve high levels of consensus in round one suggests participants felt that these actions were not controversial, though different views may have been obtained by suggesting other aspects of care. We also recognise that the respondent numbers were small in comparison to the overall BASP and NSNF membership, and there was no similar society used to contact other AHPs. Further, we did not seek the opinions of GPs themselves and what their role would be in the management of stroke patients with cognitive impairments. However, we wished to focus on the six-month review and the majority of respondents had first-hand experience to guide their views about priorities during patient care at this time interval. We do appreciate that other professionals are involved in the six-month review, whom are not represented in this sample. This spread was limited by the member organisations we approached but we did still find that over half of the participants in each round conducted the six-month review. However, over 50% of participants in round one had 11 or more years of clinical experience with around two thirds of participants in round two having 11 or more years of clinical experience. This is important because a Delphi survey looks to seek consensus opinion from a group of experts [22], which we believe has been obtained here in spite of the relatively modest numbers who responded. Further, given the high level of consensus in round one it would seem unlikely that an alternative pattern would have been found amongst larger numbers of respondents. We do recognise that the use of only two rounds limited the conclusions to the strongest agreement only. Additional rounds would be needed to understand movement across final lower rankings and confirm the stability of the rankings. Further, the small number of respondents makes the data exploratory rather than definitive, and a larger study would be needed to confirm that the results are generalisable. We did not seek the opinions of other groups who would have greater assessment expertise in this area for example psychologists, as we wanted to capture the views from the clinicians involved in initially identifying issues amongst the wider stroke population. Mental health practitioners provide very detailed assessments to clarify exact nature of broader cognitive problems and do not perform the 6 month reviews. Where available, they receive referrals once an issue has been identified but there remains very inadequate provision of psychological services nationally, and if this professional group had been included it would have been difficult to interpret the data for implications across all services. Our project was exploring the initial step prior to their expert involvement as part of a wider holistic review.

### Clinical implications

The results indicate that in line with national recommendations, professionals would value improvements in the care of patients with cognitive difficulties after stroke. National Clinical Guidelines recommend routine review of cognition alongside assessment and management of mood during the rehabilitation phase post-stroke [9, 12]. The importance of cognitive care is recognised by professionals in this study but is not reflected in the way services are commissioned and provided. A Care Quality Commission Review in 2011 found that less than 40% of areas provided access to psychological therapy for all stroke-survivors regarding their cognitive difficulties [23]. Upon transfer home, the review also found that improvement was needed on the information supplied to stroke-survivors and carers regarding stroke-related cognitive problems such as memory and concentration [23]. An audit of longer term stroke services found that the longest delays in waiting times were found in accessing psychological support, particularly when compared with support for aspects of physical health e.g. physiotherapy [24]. This is in spite of recommendations that commissioning stroke services should be the same for physical care, rehabilitation as well as psychological care [25]. Six-month reviews enable clinicians and stroke patients to highlight these post-stroke cognitive deficits. However, there are still a number of areas where 6 month reviews are not being consistently performed [24]. This limits the opportunities for stroke patients and carers to present to their clinical provider with the more silent symptoms post-stroke such as cognition and subsequently accessing help in this area. Further, it is not quite clear who should be providing long-term care for post-stroke cognitive deficits.

Our study found that when considering the actions required at the 6-month review, clinicians believe that access to psychology is a key part of ongoing care in the general stroke population (case 1) and the young stroke-survivors (case 2). The role of primary care for cognitive assessment was generally not agreed. Currently, it seems that this gap in specialist psychological services may be met by staff in non-psychology disciplines (e.g. occupational therapists) who have voiced concerns about growing responsibilities associated with psychological assessments and the lack of necessary skills and training in this area [26]. If psychological services remain inconsistent across areas, then suitable alternative options should be found. An example of one in-patient initiative involved a “skill mix model” in order to maximise the resources available to the service and meet patient needs [27] by stratifying clinical psychology support at different levels of intervention [27]. In future, this may well also involve upskilling community psychology services or training other non-psychology disciplines within stroke care.

Memory clinics could potentially be an area where patients are followed up particularly if they demonstrate significant cognitive deficits post-stroke but their role is unclear. Although direct access was generally not approved across scenarios, this opinion seemed to change when we also sought the views of clinicians on actions if a risk assessment procedure was used in a patient reporting subjective memory complaints. Risk assessment in dementia is well researched with a number of tools developed to predict future dementia in the general population [17, 18]. Some have also been developed for stroke populations for post-stroke cognitive impairment [28] and dementia [29]. However, none are currently used in clinical care as few models have been externally validated. If this approach were to be used in the context of stroke in the future, then direct access to memory clinics for those at high risk of a future dementia illness appears to be supported by clinicians in stroke services. However, memory clinics vary in terms of staffing levels and follow-up processes [30], and it is unclear whether memory clinic services would be able to manage this additional workload. Further, although the majority are able to see new patients within 6 weeks, other memory assessment service users can wait on average over 12 weeks [30]. It may not be cost-effective to refer every stroke patient with cognitive difficulties into a memory assessment service based on a single screening assessment. By ensuring access to services outside of clinical stroke care, it may be possible to address inadequacies in specialist care [16], but this must be balanced against the burden placed upon memory assessment services and the views of stroke-survivors and their families when using a risk assessment approach [31].

Compliance with secondary prevention was highly ranked by participants when discussing management of those stratified into high and low risk categories via risk assessment tools. However, trial evidence about the impact on cognition has been mixed. When patients with recent stroke had intensive blood pressure and lipid lowering management there was no alteration in cognition at 2 years [32]. Active blood pressure treatment has previously been found to reduce risks of dementia and cognitive decline, but this was also associated with recurrent stroke with no clear effect on either dementia or cognitive decline has been found in the absence of recurrent stroke [33]. If future evidence supports specific secondary prevention measures then it is useful to understand that clinicians would support implementation.

## Conclusions

This exploratory Delphi study has described consensus for actions by clinicians at the six-month review of stroke-survivors presenting with cognitive difficulties in various contexts. There was strong support by participants in this study for improved specialist psychological support to be made available for patients. Stratification towards specialist memory services would be supported through use of risk assessment tools but further work is needed to assess the feasibility and cost-effectiveness of this approach.

## Supplementary information

**Additional file 1.** Online Supplementary Table 1: Overall Responses from Round 1.

**Additional file 2.** Online Supplementary Table 2: Overall Responses from Round 2.

## Data Availability

No further data will be made available.

## Abbreviations

BASP: British association of stroke physicians
GP: General practitioner
IQR: Interquartile range
NSNF: National stroke nursing forum
UK: United Kingdom
