# Supplementary material for: Care priorities for stroke patients developing cognitive difficulties: a Delphi survey of UK professional views
Source: BMC Health Serv Res. 2020 Aug 5;20:717. doi: 10.1186/s12913-020-05558-y (PMC7404922; doi:10.1186/s12913-020-05558-y)
Supplement: Supplementary file 1 — Additional file 1. Online Supplementary Table 1: Overall Responses from Round 1. [file 12913_2020_5558_MOESM1_ESM.docx]

**Online Supplementary Table 1: Overall Responses from Round 1**

|  | **Aggregated responses from round 1**  **n (%)** | | | | | | |
| --- | --- | --- | --- | --- | --- | --- | --- |
| **Case 1: Statement** | **very strongly disapprove (1)** | **quite strongly disapprove (2)** | **Disapprove (3)** | **Neutral (4)** | **Approve (5)** | **quite strongly approve (6)** | **very strongly approve (7)** |
| *Signposting individuals to other sources of information e.g. Stroke Association* | 0 | 0 | 0 | 1 (2) | 8 (18) | 13 (29) | 23 (51) |
| *Access to psychological services* | 0 | 1 (2) | 1 (2) | 2 (4) | 9 (20) | 7 (16) | 25 (56) |
| *Additional communication with the GP* | 0 | 0 | 0 | 7 (16) | 15 (33) | 13 (29) | 10 (22) |
| *Screening for a mood disorder* | 0 | 0 | 0 | 2 (4) | 11 (24) | 10 (22) | 22 (49) |
| *Ensuring compliance to secondary prevention is in place* | 0 | 0 | 0 | 4 (9) | 9 (20) | 10 (22) | 22 (49) |
| *Ensuring allied health professional community follow-up e.g. occupational therapist for additional follow-up review in the community* | 0 | 0 | 1 (2) | 5 (11) | 9 (20) | 11 (24) | 19 (42) |
| *Follow-up in stroke-services* | 1 (2) | 1 (2) | 2 (4) | 6 (13) | 6 (13) | 12 (27) | 17 (38) |
| *Direct access to memory clinic services* | 0 | 0 | 2 (4) | 6 (13) | 17 (38) | 11 (24) | 9 (20) |
| *Cognitive screen e.g. MoCA during six-month stroke clinic review* | 1 (2) | 0 | 2 (4) | 8 (18) | 12 (27) | 5 (11) | 17 (38) |
| *GP to perform cognitive screen following discharge from specialist services* | 3 (7) | 2 (4) | 4 (9) | 15 (33) | 12 (27) | 7 (16) | 2 (4) |
| **Case 2: Statement** |  |  |  |  |  |  |  |
| *Access to psychological services* | 0 | 1 (2) | 0 | 0 | 5 (11) | 4 (9) | 35 (78) |
| *Signposting individuals to other sources of information e.g. Stroke Association* | 0 | 0 | 0 | 1 (2) | 5 (11) | 9 (20) | 30 (67) |
| *Screening for a mood disorder* | 0 | 0 | 0 | 1 (2) | 9 (20) | 6 (13) | 29 (64) |
| *Ensuring compliance to secondary prevention is in place* | 0 | 0 | 0 | 1 (2) | 9 (20) | 9 (20) | 26 (58) |
| *Ensuring allied health professional community follow-up e.g. occupational therapist for additional follow-up review in the community* | 1 (2) | 0 | 0 | 3 (7) | 5 (11) | 10 (22) | 26 (58) |
| *Additional communication with the GP* | 0 | 0 | 0 | 4 (9) | 14 (31) | 9 (20) | 18 (40) |
| *Follow-up in stroke-services* | 0 | 1 (2) | 1 (2) | 3 (7) | 8 (18) | 11 (24) | 21 (47) |
| *Cognitive screen e.g. MoCA during six-month stroke clinic review* | 1 (2) | 0 | 2 (4) | 6 (13) | 6 (13) | 8 (18) | 22 (49) |
| *GP to perform cognitive screen following discharge from specialist services* | 1 (2) | 3 (7) | 3 (7) | 13 (29) | 16 (36) | 3 (7) | 6 (13) |
| *Direct access to memory clinic services* | 1 (2) | 0 | 1 (2) | 7 (16) | 16 (36) | 7 (16) | 13 (29) |
| **Case 3: Statement** |  |  |  |  |  |  |  |
| Signposting individuals to other sources of information e.g. Stroke Association | 0 | 0 | 0 | 2 (4) | 9 (20) | 11 (24) | 23 (51) |
| Screening for a mood disorder | 0 | 0 | 0 | 2 (4) | 12 (27) | 9 (20) | 22 (49) |
| Ensuring compliance to secondary prevention is in place | 0 | 0 | 1 (2) | 2 (4) | 11 (24) | 12 (27) | 19 (42) |
| Ensuring allied health professional community follow-up e.g. occupational therapist for additional follow-up review in the community | 0 | 0 | 1 (2) | 4 (9) | 11 (24) | 9 (20) | 20 (44) |
| Follow-up in stroke-services | 0 | 3 (7) | 3 (7) | 8 (18) | 7 (16) | 10 (22) | 14 (31) |
| Access to psychological services | 0 | 2 (4) | 2 (4) | 4 (9) | 15 (33) | 12 (27) | 10 (22) |
| Additional communication with the GP | 0 | 0 | 1 (2) | 6 (13) | 16 (36) | 6 (13) | 16 (36) |
| Direct access to memory clinic services | 2 (4) | 0 | 3 (7) | 10 (2) | 16 (36) | 5 (11) | 9 (20) |
| Cognitive screen e.g. MoCA during six-month stroke clinic review | 1 (2) | 1 (2) | 1 (2) | 14 (31) | 6 (13) | 11 (24) | 11 (24) |
| GP to perform cognitive screen following discharge from specialist services | 2 (4) | 1 (2) | 7 (16) | 14 (31) | 14 (31) | 5 (11) | 2 (4) |
| **Case 4: Statement** |  |  |  |  |  |  |  |
| Ensuring compliance to secondary prevention is in place | 0 | 0 | 0 | 1 (2) | 6 (13) | 12 (27) | 26 (58) |
| Direct access to memory clinic services | 0 | 0 | 0 | 4 (9) | 6 (13) | 12 (27) | 23 (51) |
| Screening for a mood disorder | 0 | 0 | 0 | 0 | 8 (18) | 15 (33) | 22 (49) |
| Signposting individuals to other sources of information e.g. Stroke Association | 0 | 0 | 0 | 2 (4) | 9 (20) | 12 (27) | 22 (49) |
| Cognitive screen e.g. MoCA during six-month stroke clinic review | 0 | 1 (2) | 1 (2) | 2 (4) | 7 (16) | 12 (27) | 22 (49) |
| Access to psychological services | 0 | 0 | 0 | 2 (4) | 12 (27) | 10 (22) | 21 (47) |
| Additional communication with the GP | 0 | 0 | 1 (2) | 2 (4) | 10 (22) | 11 (24) | 21 (47) |
| Ensuring allied health professional community follow-up e.g. occupational therapist for additional follow-up review in the community | 0 | 0 | 0 | 7 (16) | 11 (24) | 9 (20) | 18 (40) |
| GP to perform cognitive screen following discharge from specialist services | 1 (2) | 1 (2) | 2 (4) | 7 (16) | 13 (29) | 12 (27) | 9 (20) |
| Follow-up in stroke-services | 1 (2) | 1 (2) | 3 (7) | 8 (18) | 10 (22) | 11 (24) | 11 (24) |
| **Case 5: Statement** |  |  |  |  |  |  |  |
| Signposting individuals to other sources of information e.g. Stroke Association | 0 | 0 | 0 | 2 (4) | 13 (29) | 9 (20) | 21 (47) |
| Ensuring compliance to secondary prevention is in place | 0 | 0 | 0 | 3 (7) | 9 (20) | 11 (24) | 22 (49) |
| Screening for a mood disorder | 0 | 0 | 0 | 3 (7) | 14 (31) | 14 (31) | 14 (31) |
| Additional communication with the GP | 0 | 0 | 0 | 9 (20) | 20 (44) | 10 (22) | 6 (13) |
| GP to perform cognitive screen following discharge from specialist services | 1 (2) | 1 (2) | 4 (9) | 8 (18) | 26 (58) | 2 (4) | 3 (7) |
| Cognitive screen e.g. MoCA during six-month stroke clinic review | 0 | 1 (2) | 2 (4) | 6 (13) | 15 (33) | 7 (16) | 14 (31) |
| Follow-up in stroke-services | 0 | 1 (2) | 7 (16) | 7 (16) | 14 (31) | 9 (20) | 7 (16) |
| Access to psychological services | 0 | 0 | 3 (7) | 13 (29) | 12 (27) | 12 (27) | 5 (11) |
| Ensuring allied health professional community follow-up e.g. occupational therapist for additional follow-up review in the community | 0 | 0 | 2 (4) | 16 (36) | 9 (20) | 7 (16) | 11 (24) |
| Direct access to memory clinic services | 0 | 0 | 4 (9) | 16 (36) | 13 (29) | 10 (22) | 2 (4) |
