# Supplementary material for: Care priorities for stroke patients developing cognitive difficulties: a Delphi survey of UK professional views
Source: BMC Health Serv Res. 2020 Aug 5;20:717. doi: 10.1186/s12913-020-05558-y (PMC7404922; doi:10.1186/s12913-020-05558-y)
Supplement: Supplementary file 2 — Additional file 2. Online Supplementary Table 2: Overall Responses from Round 2. [file 12913_2020_5558_MOESM2_ESM.docx]

**Online Supplementary Table 2: Overall Responses from Round 2**

| **Case 1** | **Total** | **Case 2** | **Total** | **Case 3** | **Total** | **Case 4** | **Total** | **Case 5** | **Total** |
| --- | --- | --- | --- | --- | --- | --- | --- | --- | --- |
| Access to psychological services | 154 | Access to psychological services | 162 | Ensuring allied health professional community follow-up e.g. occupational therapist for additional follow-up review in the community | 155 | Direct access to memory clinic services | 151 | Screening for a mood disorder | 160 |
| Screening for a mood disorder | 145 | Ensuring allied health professional community follow-up e.g. occupational therapist for additional follow-up review in the community | 147 | Screening for a mood disorder | 146 | Cognitive screen e.g. MoCA during six-month stroke clinic review | 149 | Signposting individuals to other sources of information e.g. Stroke Association | 154 |
| Ensuring allied health professional community follow-up e.g. occupational therapist for additional follow-up review in the community | 129 | Screening for a mood disorder | 144 | Follow-up in stroke-services | 143 | Ensuring compliance to secondary prevention is in place | 143 | Ensuring compliance to secondary prevention is in place | 142 |
| Additional communication with the GP | 122 | Cognitive screen e.g. MoCA during six-month stroke clinic review | 119 | Access to psychological services | 125 | Screening for a mood disorder | 139 | Cognitive screen e.g. MoCA during six-month stroke clinic review | 116 |
| Signposting individuals to other sources of information e.g. Stroke Association | 115 | Follow-up in stroke-services | 117 | Signposting individuals to other sources of information e.g. Stroke Association | 117 | Access to psychological services | 122 | Follow-up in stroke-services | 112 |
| Cognitive screen e.g. MoCA during six-month stroke clinic review | 110 | Ensuring compliance to secondary prevention is in place | 115 | Ensuring compliance to secondary prevention is in place | 115 | Additional communication with the GP | 104 | Additional communication with the GP | 109 |
| Follow-up in stroke-services | 107 | Signposting individuals to other sources of information e.g. Stroke Association | 108 | Additional communication with the GP | 105 | Ensuring allied health professional community follow-up e.g. occupational therapist for additional follow-up review in the community | 98 | Access to psychological services | 100 |
| Direct access to memory clinic services | 106 | Additional communication with the GP | 99 | Cognitive screen e.g. MoCA during six-month stroke clinic review | 96 | Signposting individuals to other sources of information e.g. Stroke Association | 91 | Ensuring allied health professional community follow-up e.g. occupational therapist for additional follow-up review in the community | 99 |
| Ensuring compliance to secondary prevention is in place | 104 | Direct access to memory clinic services | 73 | Direct access to memory clinic services | 94 | Follow-up in stroke-services | 80 | GP to perform cognitive screen following discharge from specialist services | 87 |
| GP to perform cognitive screen following discharge from specialist services | 63 | GP to perform cognitive screen following discharge from specialist services | 71 | GP to perform cognitive screen following discharge from specialist services | 59 | GP to perform cognitive screen following discharge from specialist services | 78 | Direct access to memory clinic services | 76 |
